# Gastric intramural hematoma subsequent to thoracic aortic dissection: Case report and literature review

**DOI:** 10.1016/j.amsu.2018.09.026

**Published:** 2018-09-27

**Authors:** Alana Costa Borges, Marcelo de Sousa Cury, Gilberto F. de Carvalho, Stella Maria Torres Furlani

**Affiliations:** a Zilda Arns Hospital and Maternity, Gastrointestinal Endoscopy, 145 Lineu Machado Av, 60520-100, Fortaleza, CE, Brazil; b SCOPE Gastrointestinal Endoscopy Unit, Gastrointestinal Endoscopy, 1148 Maracaju St, 79002-212, Campo Grande, MS, Brazil; c Zilda Arns Hospital and Maternity, Radiology, 145 Lineu Machado Av, 60520-100, Fortaleza, CE, Brazil; d César Cals General Hospital, Gastrointestinal Endoscopy, 545 Imperador Av, 60015-152, Fortaleza, CE, Brazil

**Keywords:** Case report, Intramural, Stomach, Hematoma, Aortic dissection

## Abstract

**Introduction:**

Intramural hematomas of the gastrointestinal tract are uncommom, usually located in the esophagus or duodenum, with idiophatic or secondary causes. We present a very rare case of gastric intramural hematoma caused by an unpublished etiology, with literature review.

**Case presentation:**

An elderly woman suffered acute thoracic aorta dissection followed by gastric intramural hematoma, diagnosed through endoscopy and computed tomography angiography. The treatment included endovascular aortic repair and conservative management.

**Discussion:**

The postulated mechanism for the bleeding in gastric intramural hematoma is shredding of terminal arteries at the point of penetration into the muscular layer with subsequent dissection of the muscularis propria from the submucosa. The most frequently cited risk factor is hemorrhagic diathesis/anticoagulant use and the main etiologies are trauma and post-interventional endoscopy. In the diagnosis work-up, computed tomography is the method of choice, usually associated with endoscopy. There is no standard of care for such rare condition. Thus, treatment may be cause-dependent, ranging from conservative to minimally invasive and/or surgery.

**Conclusions:**

Gastric intramural hematoma is a rare disorder with many causes and we described a new etiology for it. The computed tomography is the diagnostic modality of choice, with the aid of other examinations. The treatment comprises conservative measures, minimally invasive approach or most commonly surgery.

## Highlights

• Gastric intramural hematoma is very rare.
• It is important to be familiarized with it, since it has so many different causes.
• This article presents a new cause for gastric intramural hematoma.
• Treatment may be conservative, minimally invasive or surgical.

## 1 Introduction

Intramural hematomas of the gastrointestinal tract are uncommom, mostly located in the esophagus or duodenum, being of idiophatic origin or secondary to a condition or intervention. A gastric intramural hematoma (GIH) is very rare, with only few previous case reports [1]. A very unusual case of GIH is presented, with literature review. The current work has been reported in line with the SCARE criteria [2].

### 1.1 Case report

An 87-year-old woman presented to the Emergency Room (ER) complaining of an acute (few hours) and intense thoracic pain radiating to the dorsum and upper abdomen, associated with nausea, dyspnea, sudoresis and hypertensive peak. She had a medical history of hypertension. Eight years ago, she underwent a hip arthroplasty, complicated by pulmonary thromboembolism (PTE), when she was started on anticoagulants for the first six months and then on aspirin associated with cilostazol daily.

On the ER's initial evaluation, chest radiograph revealed an enlarged mediastinum. The patient had persisting pain, despite the administration of morphine and nytroglicerin, without hemodynamic instability. She had normal cardiac enzymes and electrocardiogram with elevated D-dimer test (8.02μg/mL). The emergency thoracic computed tomography angiography (CTA) showed an ectatic ascending aorta (4.0cm), descending aorta aneurysm with mural thrombus and laminar mediastinal fluid, excluding PTE. The echocardiogram identified in addition to the aortic ectasia, a mild aortic insufficiency. Subsequently, the patient was admitted to the hospital and transferred to the Intensive Care Unit (ICU).

As the pain subsided, she was managed conservatively. After three days, she had another burst of severe epigastric pain, associated with hematemesis, hemoglobin drop to 7.9g/dL (previously 11.9g/dL), hypotension (80 × 40 mmHg) and tachycardia (113 bpm).

**Fig. 1:**
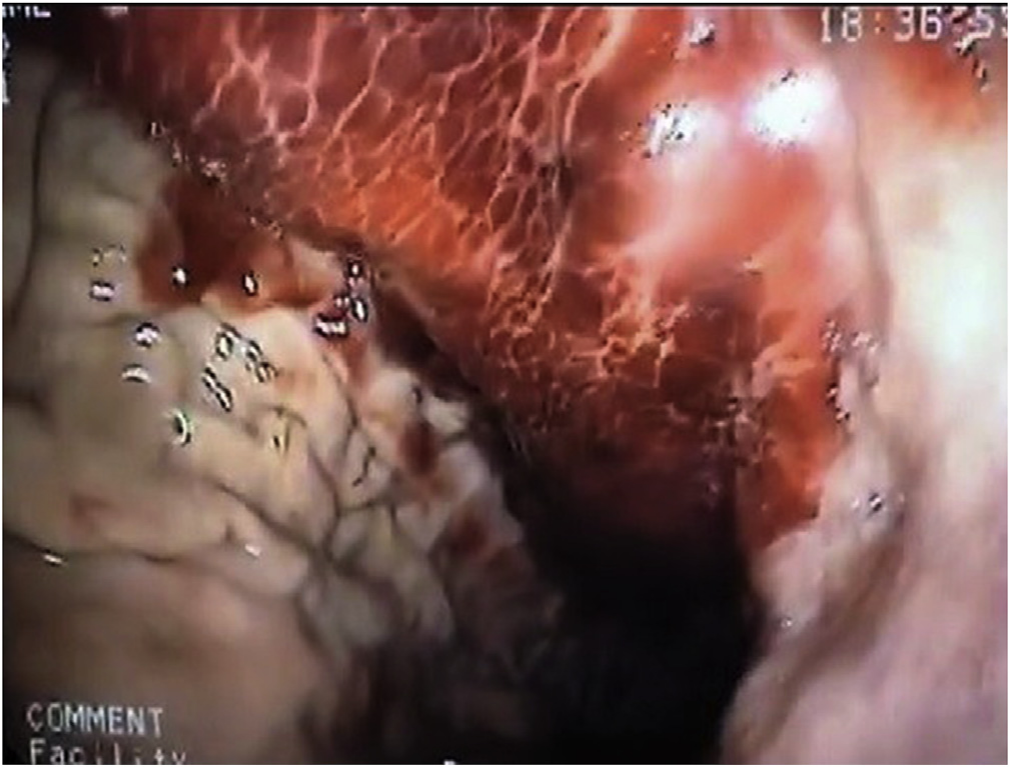
Large intramural gastric hematoma occupying the corpus on endoscopy.

**Fig. 2:**
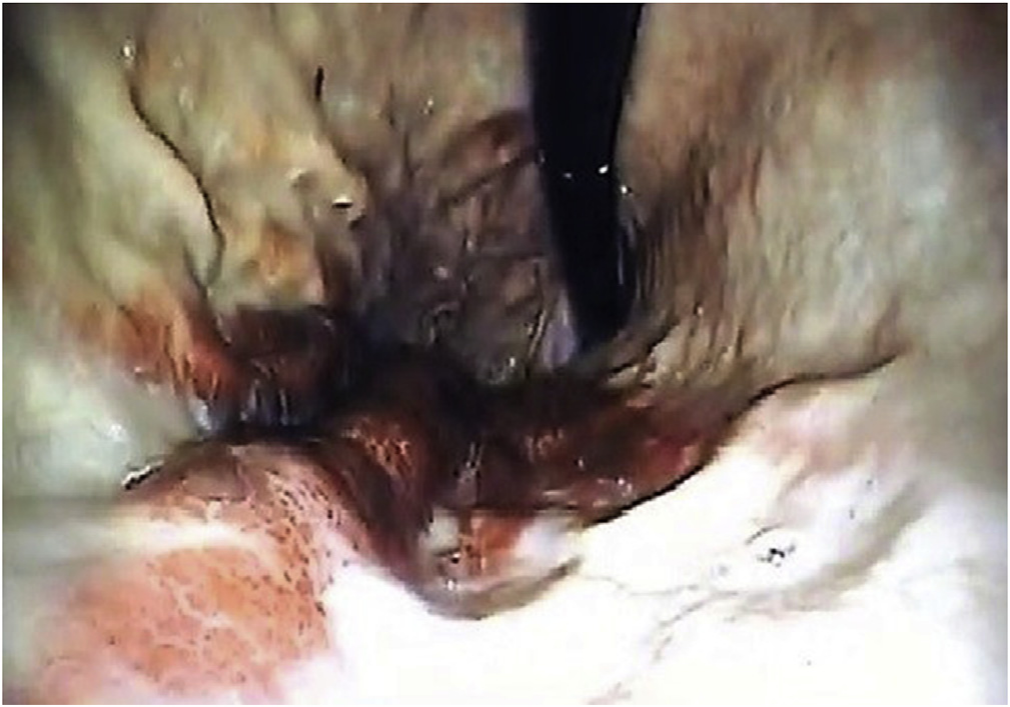
Large intramural hematoma in a retroflexed endoscopic view.

The standard protocol of care of patients with acute upper gastrointestinal bleeding was executed: NPO diet, intravenous fluids, blood transfusion (which was necessary in this case) and proton pump inhibitors (PPI), followed by esophagogastroduodenoscopy (EGD). The EGD diagnosed an enormous bright red subepithelial mass, occupying the fundus and corpus in the lesser curvature, with luminal bulging, no mucosal bleeding, nor ulcerations/erosions, as shown in Fig. 1, Fig. 2. No therapy nor biopsies were performed. The remainder of the stomach, esophagus and duodenum were normal.

The patient denied any recent trauma, surgery or endoscopic intervention. Hence, she underwent further investigation in order to determine the nature of the hematoma, with the working differential diagnoses of intramural neoplasia and dissecting visceral aneurysm.

**Fig. 3:**
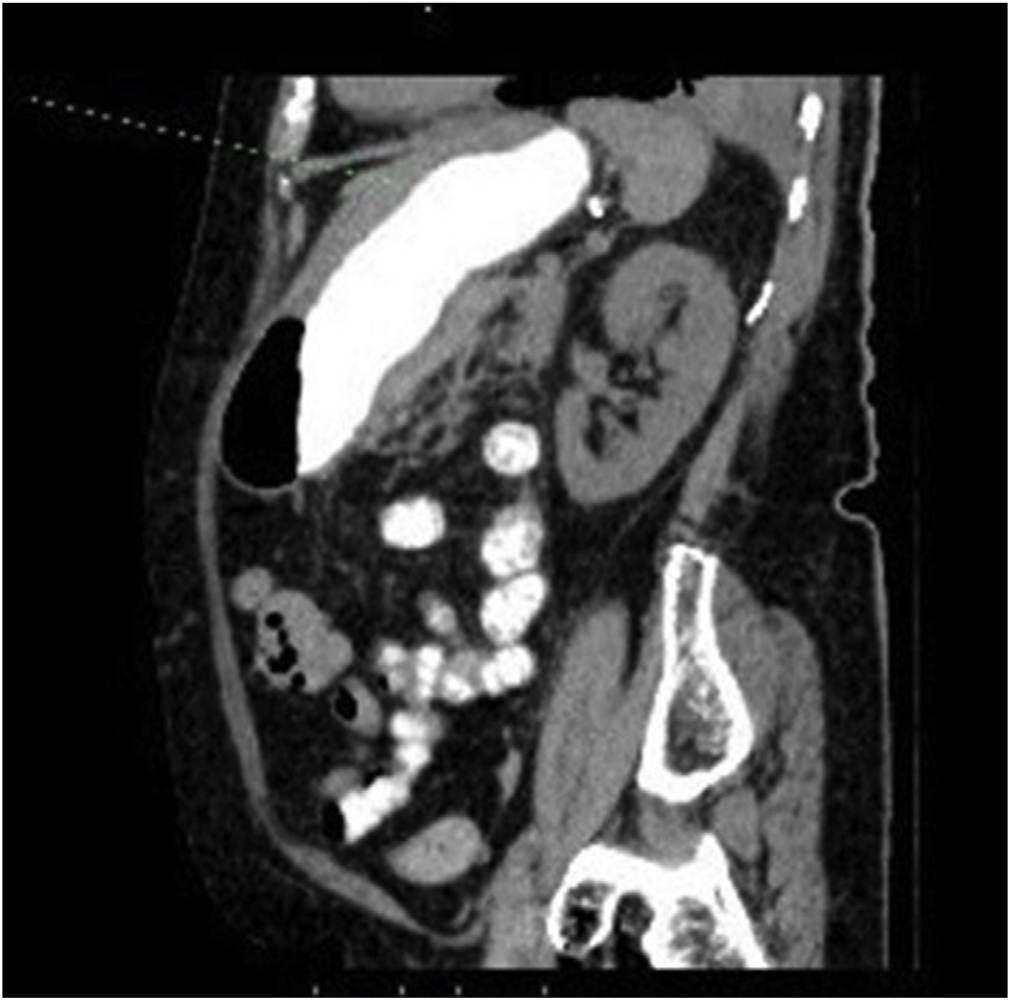
Parietal hematoma of the corpus and fundus on computed tomography.

**Fig. 4:**
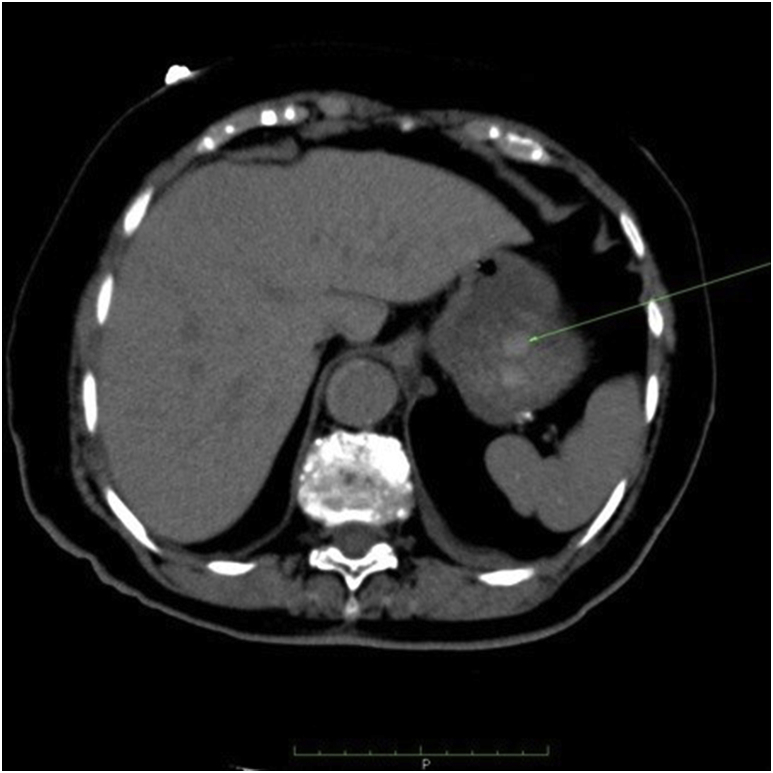
Gastric hematoma with a small amount of luminal blood on computed tomography.

**Fig. 5:**
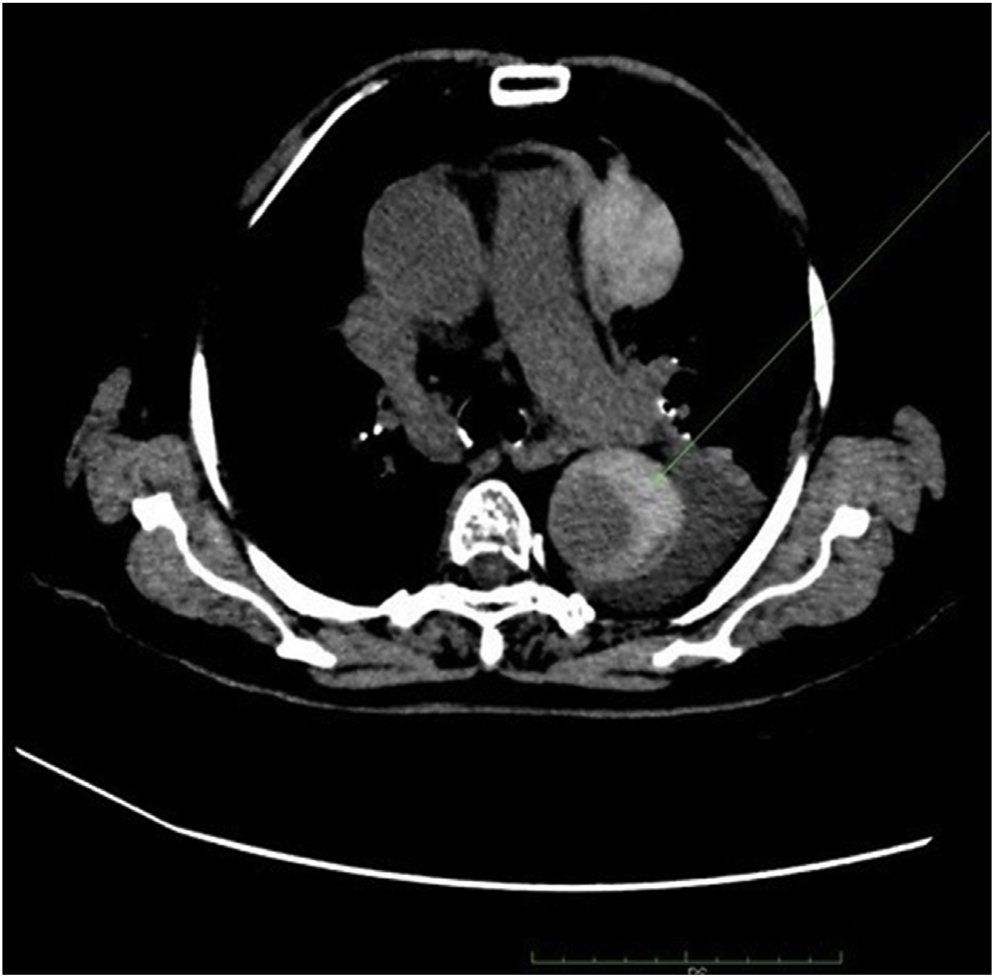
Hyperdense crescent-like aortic wall hematoma on computed tomography.

After the EGD, she was submitted to a new CTA, with evidence of a slightly hyperdense wall thickening of the corpus (4.0cm) and fundus (2.5cm), a small amount of blood compatible material in the lumen and perigastric adipose tissue hyperdensity, as shown in Fig. 3, Fig. 4. It also showed a descending aorta aneurysm with an extensive (from the subclavian origin to the thoraco-abdominal transition) crescent-like mural thrombus, which was hyperdense in the non-enhanced scan, with intimal flap and false lumen, suggesting dissecting acute intramural aortic hematoma, as demonstrated in Fig. 5. There were signs of rupture, characterized by the presence of mediastinal hematoma measuring 6.4 × 5.4 × 3.3cm at the level of the pulmonary trunk and left pleural effusion. The conjoint findings made the final diagnosis of aortic dissection with contained rupture.

Hematologic examination found no other coagulopathies and the only predisposing factor was the previous use of two antithrombotic antiplatelet drugs, which were suspended since the admission. Thus, the aortic dissection with contained rupture was considered the cause for the GIH.

Endovascular repair was performed, with implant of an aortic graft. The angiography presented no contrast extravasation of the gastroparietal branch, nor a visceral aneurysm. In the next day, control EGD revealed the GIH was stable, with slight regression. The oral diet was resumed on the second post-operative day. The GIH was managed conservatively and the patient had favorable outcome.

Approximately three weeks after the diagnosis, another EGD showed GIH involution. She was discharged after a few days and the prescription of aspirin was restarted. On routine follow-up, on the 30th postoperative day, she was submitted to a control Endoscopic Ultrasound (EUS), which was normal, the GIH had disappeared. The patient remains on follow-up and regular appointments with her vascular surgeon and geriatrician. The patient gave consent for publication of her case in the medical literature.

## 2 Discussion

GIH is a very rare condition [3]. It can arise in the submucosal or muscular layer of the organ [4]. The postulated mechanism for the bleeding is shredding of terminal arteries at the point of penetration in the muscular layer with subsequent dissection of the muscularis propria from the submucosa [5,6] and the most common contributing factor is hemorrhagic diathesis/anticoagulant use [7].

An extensive Pubmed search was conducted, looking for all reported data in adult patients in the English literature and utilizing the following entries [gastric intramural hematoma], [hematoma gastric wall], [gastric subepithelial hematoma] and [gastric subserosal hematoma], with the findings summarized in Table 1.

**Table 1:** Published literature on gastric intramural hematoma.

Causes	Reference	N	Management					
			Surgery	Conservative	TAE	Endoscopy	CT	NA
Trauma	2, 12	33	22	11				
Coagulopathy	1, 3, 4, 7, 8, 28	6		4	1			1
Spontaneous	9, 10,11, 24, 27	5	3	2				
Endoscopy	5, 6, 13, 14, 15, 16, 17,18	8	2	3	1	2		
Pancreatitis	22, 23	2		1			1	
Peptic Ulcer	20	1	1					
Amyloidosis	21	1	1					
Foreign Body	19	1		1				
**Total**	26	57	29	22	2	2	1	1

TAE – Transcatheter Arterial Embolization, CT – Computed Tomography, NA - Not applicable.

In the review, with a total of 26 articles and 57 patients, the main etiologies were trauma, post-interventional endoscopy, coagulopathy/anticoagulants and spontaneous [3,[8], [9], [10], [11], [12]]. Gastric blunt trauma is infrequent, with a reported incidence up to 1.7%. It may occur in high velocity impact involving the epigastrium in the post-prandial period, due to tangential tearing along fixed points, increased intraluminal pressure and crushing against the vertebral bodies. The most frequently injured part of the stomach is the fundus, followed by the corpus [13].

The endoscopy-related cases include post-percutaneous endoscopic gastrostomy (PEG), argon plasma coagulation (APC), endoscopic mucosal resection (EMR), endoscopic submucosal dissection (ESD), EUS-guided fine needle aspiration (EUS-FNA) and post-injection therapy [6,7,[14], [15], [16], [17], [18], [19]]. Furthermore, there are few reports with anedoctal causes for GIH, such as foreign body, peptic ulcer disease, amyloidosis and pancreatitis [[20], [21], [22], [23], [24]]. The authors did not find any previous reports like the present, with aortic dissection-associated GIH.

In GIH work-up, CT is the current diagnostic tool of choice [1]. Solazzo et al. [13], in a very elegant study on gastric blunt trauma, proposed a radiological-based grading system with diagnostic and prognostic applications. The grades range from 1 to 4, representing gastric contusion to full thickness rupture. The authors believe such classification can be extrapolated for the present case, despite of the different cause. Thus, our patient would be classified as grade 1 lesion, with the suggestion of conservative care, which was performed, with the additional correction of the aortic dissection/rupture.

Endoscopy plays a key role in the investigation of GIH, especially when it is post-interventional endoscopy. Usually, there is gastric luminal narrowing and a submucosal bright-red or dark-red mass, depending on the examination's timing, sometimes, with the presence of active mucosal bleeding [4]. EUS is also a useful method to assess lymph nodal status and the depth of the mass, providing cytological and histological material, to differentiate from the subephitelial tumors/GISTs [3,25]. In this case, EUS was undertaken during outpatient follow-up to assess regression of the hematoma and perform EUS-FNA if needed.

However, there is the remote possibility of not making a final diagnosis in time. The only GIH-attributed fatality in the literature happened in an elderly man. It was consequent to the rupture of a left gastric dissecting aneurysm, in the so-called “double-rupture phenomenon”, i.e. delayed fatal rupture of a visceral splanchnic aneurysm [26], when they first hemorrhage into the lesser sac with subsequent overflow into the peritoneal cavity, followed by sudden circulatory collapse and estimated mortality rate of 70% [27].

### 2.1 Management of GIH

There is no standard of care for such rare condition. The treatment has a progressive spectrum. It can be conservative, resort to minimally invasive therapies (angiography, percutaneous drainage and endoscopic treatment) and/or to surgery [4,[13], [14], [15], [16],^18^,^23^,^28^].

The treatment may be cause-dependent. Hence, GIHs secondary to coagulopathy are generally managed conservatively with blood transfusion and anticoagulation reversal [1,29]. Oral intake is usually interrupted in patients with abdominal pain, acute bleeding and symptoms of gastric outlet oclusion [5].

If there is active bleeding or a trend toward enlargement, transcatheter arterial embolization (TAE) may be indicated. However, it is only technically possible if contrast extravasation of the gastroparietal branch of the left gastric artery is identified [4,16,29].

There are two documented cases of post-acute pancreatitis GIHs. One was treated conservatively and the other resorted to CT-guided percutaneous drainage successfully [23,24].

In the interventional endoscopy-related GIHs, endoscopic approach may be attempted, with two successful reports. In the first, in an APC-caused hematoma, the authors performed an endoscopic incision and drainage [14]. The second GIH, which was consequential to an EUS-FNA, was treated with endoscopic band ligation in the bleeding spot [18]. Furthermore, Park et al. [16] described a post-EMR GIH with combined therapeutics: endoscopic hemocliping in the resection wound and subsequenty TAE.

In traumatic GIHs, therapy is tailored, considering the patient's clinical status and prognostic stratification, to decide between close follow-up or surgery. In Solazzo et al.'s case series, the biggest one to date so far, most of the patients (68.7%) were treated surgically [13].

Surgery is still the most frequently employed treatment. As exposed in the current review, 50.8% of the sample underwent surgical exploration. It is especially recommended in cases with unclear diagnosis, suspected complications, ongoing bleeding and failure of minimally invasive therapy [5,25,28,29]. The decision to operate emergently is largely driven by the clinical scenario [6].

## 3 Conclusion

Gastric Intramural hematoma is rare and has many different causes. This article described a new etiology for it – aortic dissection with contained rupture favoured by the combined use of two antiplatelet drugs. The computed tomography is the diagnostic modality of choice, with the aid of other examinations, as performed in this case. The treatment comprises conservative measures, minimally invasive approach or most commonly surgery. Learning from this case, physicians should be aware of this rare finding and prepare to make a faster diagnosis and consequential treatment.

## Ethical approval

This article is exempt from ethical approval. The patient gave consent for publication of her case in the medical literature.

## Sources of funding

This article received no funding.

## Author contribution

Borges AC: management of the patient, article concept, data collection and manuscript writing;

Cury MS: interpretation of data and images, analysis and discussion;

Carvalho GF: technical review of the article and imagiological validation of the findings;

Furlani SMT: final review of the article.

## Conflicts of interest

All the authors declare no conflicts of interest.

## Research registration number

None.

## Guarantor

Borges AC.

## Fundings

None.

## Appendix A Supplementary data

SCARE Guideline Checklist SCARE Guideline Checklist
